# Antibiotic prophylaxis may not be necessary in patients with asymptomatic bacteriuria undergoing intradetrusor onabotulinumtoxinA injections for neurogenic detrusor overactivity

**DOI:** 10.1038/srep33197

**Published:** 2016-09-12

**Authors:** Lorenz Leitner, Ulla Sammer, Matthias Walter, Stephanie C. Knüpfer, Marc P. Schneider, Burkhardt Seifert, Jure Tornic, Ulrich Mehnert, Thomas M. Kessler

**Affiliations:** 1 Neuro-Urology, Spinal Cord Injury Center & Research, University of Zürich, Balgrist University Hospital, Zürich, Switzerland; 2 Department of Urology, University Hospital Basel, Basel, Switzerland; 3 Brain Research Institute, University of Zürich and Department of Health Sciences and Technology, Swiss Federal Institute of Technology Zürich, Zürich, Switzerland; 4 Epidemiology, Biostatistics and Prevention Institute, Department of Biostatistics, University of Zürich, Zürich, Switzerland

## Abstract

Many of the patients undergoing intradetrusor onabotulinumtoxinA injections for refractory neurogenic detrusor overactivity (NDO) present with chronic bacteriuria. In these patients, antibiotic prophylaxis has been widely recommended since bacteriuria might impair treatment efficacy and cause urinary tract infections (UTI) but the evidence is limited. The aim of this study was to evaluate if an antibiotic prophylaxis is needed in patients with asymptomatic bacteriuria undergoing intradetrusor onabotulinumtoxinA injections. Between 06/2012 and 12/2014, a consecutive series of 154 patients undergoing a total of 273 treatment cycles were prospectively evaluated. Before treatment urine samples were collected, patients with no clinical signs for UTI underwent onabotulinumtoxinA injections, no antibiotic prophylaxis was given. Asymptomatic bacteriuria was found in 73% (200/273 treatments). Following treatment, UTI occurred in 5% (9/200) and 7% (5/73) of patients with and without bacteriuria, respectively. Intradetrusor onabotulinumtoxinA injections were clinically and urodynamically successful in 70% (192/273). There was no association between bacteriuria and treatment-related adverse events (odds ratio 0.64, 95% CI 0.23–1.81, p = 0.4) nor between bacteriuria and therapy failure (odds ratio 0.78, 95% CI 0.43–1.43, p = 0.4). Thus, we conclude that antibiotic prophylaxis needs to be critically reconsidered in patients undergoing intradetrusor onabotulinumtoxinA injections, especially taking into account the alarming antibiotic resistance worldwide.

A wide range of neurological disorders may cause neuro-urological symptoms^1^. According to the Guidelines on Neuro-Urology (http://uroweb.org/guideline/neuro-urology/) of the European Association of Urology (EAU), lower urinary tract function is impaired in up to 95% of patients with spinal cord injury (SCI)^2^, up to 96% of patients with spina bifida^3^ and in almost all patients suffering from multiple sclerosis for more than 10 years^4^. Asymptomatic bacteriuria is common in these patients (23–89% if preforming intermittent self-catheterisation and up to 100% if reliant on an indwelling catheter), mainly triggered by some type of catheterisation as a result of neurogenic lower urinary tract dysfunction^5^. In patients with neurogenic detrusor overactivity (NDO)^1^, major concerns are high intravesical pressures, which may lead to end stage renal failure, urinary incontinence relevantly impairing quality of life and recurrent urinary tract infections (UTI) with an incidence of 29–36%^6^.

Intradetrusor onabotulinumtoxinA injections are generally accepted as a highly effective, minimally invasive and well-tolerated therapy for patients suffering from refractory NDO^1^,^7^,^8^. However, there is no consensus on the ideal technique of injection, dosage and optimal time intervals until repeated injections. Moreover, the exact mechanism of action and long-term effects remain to be elucidated^9^,^10^.

As bacteriuria might impair efficacy and cause UTI, antibiotic prophylaxis is a widely-used practice in patients undergoing intradetrusor onabotulinumtoxinA injections. Nevertheless, the evidence is very limited and there is a lack of specific recommendations in the guidelines of the EAU^11^,^12^ and the Infectious Diseases Society of America Guidelines (IDSA)^5^,^13^.

Therefore, the aim of the present study was to evaluate if antibiotic prophylaxis is needed in patients with asymptomatic bacteriuria undergoing intradetrusor onabotulinumtoxinA injections for refractory NDO.

## Patients and Methods

### Patients

From June 2012 to December 2014, a consecutive series of 154 patients, undergoing a total of 273 intradetrusor onabotulinumtoxinA injections for NDO were prospectively evaluated at the Spinal Cord Injury Center, Balgrist University Hospital, Zürich, Switzerland. Study exclusion criteria were current UTI (defined by the presence of ≥10^3^ colony forming units/mL (CFU) in urine culture and at least one of the following symptoms/signs not explained by any other cause: fever > 38 °C, intensification of pain in the bladder/lower back, intensification of spasticity, intensification of lower urinary tract symptoms) and age < 18 years. The study including all experimental protocols were approved by the local ethics committee (Kantonale Ethikkommission Zürich) and is registered with ClinicalTrials.gov (study registration number: NCT01293110). Informed consent was obtained from all subjects. Methods were carried out in accordance with the relevant guidelines. All definitions and units are according to the standards recommended by the International Continence Society^14^.

### Investigations and treatment

All subjects underwent neuro-urological assessment^1^ and urodynamic investigation (UDI) according to good urodynamic practices following the recommendations of the International Continence Society^15^,^16^. If applicable for intradetrusor onabotulinumtoxinA injections, patients were enrolled into the study. Before treatment, urine samples were collected by sterile catheterisation for urine culture. In accordance with previously published guidelines, asymptomatic bacteriuria was defined by the presence of ≥10^2^ CFU^5^,^11^ in urine specimens of patients without symptoms/signs (as described above) referable to UTI^5^. Patients with no symptoms/signs for UTI underwent intradetrusor onabotulinumtoxinA injections without antibiotic prophylaxis. Patients with UTI were excluded and adequately treated according to the antibiotic sensitivity pattern. 6 weeks after treatment, neuro-urological assessment and UDI were repeated.

Intradetrusor onabotulinumtoxinA injections were routinely performed in local anaesthesia in an outpatient setting as described previously^17^. In brief, lidocaine gel (Instillagel^®^ 2 × 10 mL, Farco-Pharma GmbH, Germany) was instilled into the urethra and exposed for 10 minutes. Urethro-cystoscopy was performed in a lithotomy or supine position using a rigid or flexible cystoscope in women and men, respectively. Patients received 20 injections of 1 mL each (10 units onabotulinumtoxinA (Botox^®^) per mL) into the detrusor at 20 different sites (i.e. 200 units onabotulinumtoxinA in total) sparing the trigone.

### Safety and efficacy assessments

All adverse events, as defined by the International Conference on Harmonisation (ICH) Good Clinical Practice (GCP) Guidelines (E6)^18^ and International Organization for Standardization (ISO, 14155)^19^, within 6 weeks following the injections, were recorded. Efficacy was assessed by clinical and urodynamic parameters.

### Outcome measures

Outcome measures were the occurrence of treatment-related adverse events, i.e. UTI, gross haematuria and bladder pain upon follow-up UDI 6 weeks after intradetrusor onabotulinumtoxinA injections. Furthermore, improvement of clinical (i.e. urinary frequency and incontinence episodes) and urodynamic parameters (i.e. maximum cystometric capacity (MCC), maximum detrusor pressure during storage phase (pdet_max_), compliance, presence of detrusor overactivity (DO) and bladder volume at first DO) were evaluated. Successful treatment was defined as appropriate clinical (defined as urinary frequency ≤8/24 hours and reduction of incontinence episodes ≥75%) and urodynamic (defined as maximum detrusor pressure during storage phase <40 cmH_2_O and bladder compliance ≥20 mL/cmH_2_O) effect.

### Statistical analyses

Data distribution was assessed by Q-Q plots. Approximately normally distributed data were presented as mean ± standard deviation (SD), skewed data as median and interquartile range. Outcomes on safety and efficacy between patients with and without bacteriuria at injection time were compared using logistic regression analysis. To address clustering of injections within patients, logistic regression analysis was performed with safety/efficacy as a dependent variable, asymptomatic bacteriuria as independent variable and robust standard error (with patients as clusters). For clinically equivalence tests, the confidence interval (CI) inclusion rule was used and according to the current literature^20^,^21^ a delta interval of ±23%, i.e. dry rate between 41% and 88%, at a CI of 90%, was defined.

Univariate analyses were performed to identify influencing factors associated with urodynamic outcome after treatment, initially with patients as clusters. As clustering did not have any impact on outcome parameters, this was ignored for further analyses. Comparing related/unrelated samples, the paired/unpaired t test was used for approximately normally distributed data and the Wilcoxon signed rank test/Mann-Whitney U test for skewed data, respectively. For comparison of unrelated and related binary data, the chi-square test and McNemar test was used, respectively.

Statistical analyses were performed using Stata 13.1 (StataCorp, College Station, TX, USA) and IBM SPSS Statistics for Windows, Version 22.0 (IBM Corp., Armonk, NY, USA) with p < 0.05 considered statistically significant.

## Results

### Patients’ characteristics

Patients’ characteristics are shown in Table 1. The mean age at enrolment was 54 ± 18 and 51 ± 18 years for female (38%, 59/154) and male (62%, 95/154) patients, respectively. A total of 273 treatments with intradetrusor onabotulinumtoxinA injections were performed.

### Bacterial patterns

**Figure 1:**
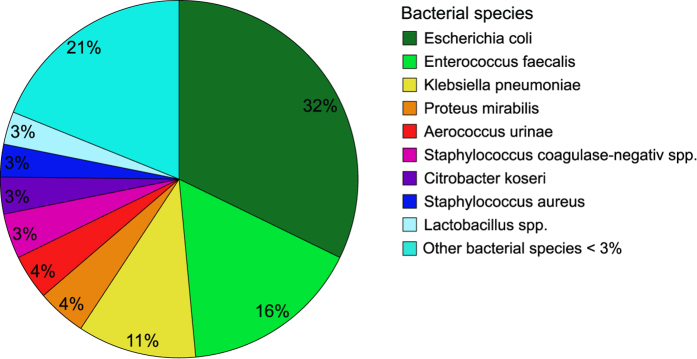
Distribution of bacterial strains. Asymptomatic bacteriuria was found in 73% (200/273 treatments). A total of 344 microorganisms and 28 different species could be isolated, including 27 different bacterial strains as well as one fungal strain (Candida albicans). Other bacterial species included (listed according to frequency): Enterobacteriaceae cloacae, Pseudomonas aeruginosa, Staphylococcus agalactiae, Proteus vulgaris, Streptococcus milleri, Serratia marcescens, Streptococcus mitis, Corynebacterium spp., Klebsiella oxytoca, Candida albicans, Streptococcus viridans, Providencia stuartii, Citrobacter freundii, Enterobacter aerogenes, Aerococcus schaalii, Morganella morganii, Stenotrophomonas maltophilia and Staphylococcus lugdunensis.

Asymptomatic bacteriuria was found in 73% (200/273 treatments). The distribution of bacterial strains is shown in Fig. 1. In 52% (104/200) of treatments with asymptomatic bacteriuria, a single bacterial strain could be isolated, a mixed bacterial pattern of 2, 3 and 4 different bacterial strains was found in 30% (60/200), 12% (24/200) and 6% (12/200) of treatments, respectively.

### Safety and efficacy

Treatment-related adverse events are presented in Table 2. UTI occurred in 7% (5/73) of cases with a sterile urine culture and in 5% (9/200) with asymptomatic bacteriuria (odds ratio 0.64, 95% CI 0.23 to 1.81, p = 0.4). One patient was hospitalized because of febrile UTI not showing bacteriuria before treatment.

Intradetrusor onabotulinumtoxinA injections were clinically and urodynamically successful in 70% (192/273). No association between asymptomatic bacteriuria and therapy failure (odds ratio 0.78, 95% CI 0.43 to 1.43, p = 0.4) was detected. The CI inclusion rule revealed a mean difference of 4.9% (90% CI -14% to 6%) in success rate, for patient with (success rate 69%) and without bacteriuria (success rate 73.9%); the CI was placed within the prior defined delta interval. The onabotulinumtoxinA effect lasted for a mean of 10 months and was not significantly (p = 0.56) different between patients with (12 ± 15 months) and without (10 ± 12 months) bacteriuria.

### Clinical and urodynamic parameters

Number of different bacterial strains, bacterial load, or method of bladder emptying, i.e. spontaneous, intermittent catheterisation or indwelling catheter, did not have any significant impact (all p ≥ 0.1) on clinical and/or urodynamic parameters.

Baseline clinical and urodynamic parameters did not differ significantly (Table 3) between patients with and without bacteriuria. A significant treatment effect regarding urinary frequency, incontinence episodes, MCC, pdet_max_, compliance, DO, and bladder volume at first DO was found in the successfully treated patients (all p < 0.001), again no significant difference between patients with and without bacteriuria could be shown (all p > 0.05).

## Discussion

### Main findings

Investigating a consecutive series of 154 patients with refractory NDO undergoing 273 intradetrusor onabotulinumtoxinA injections without antibiotic prophylaxis, safety and efficacy of the therapy could be ensured, even if asymptomatic bacteriuria was present. Within 6 weeks after treatment, UTI occurred in 5% (9/200) of the patients with asymptomatic bacteriuria pre-treatment and in 7% (5/73) of those with a sterile urine culture. The efficacy rate of 70%, i.e. appropriate clinical and urodynamic effect, was without any association between asymptomatic bacteriuria and therapy failure. In addition, there was sustained onabotulinumtoxinA effect duration of a mean of 10 months showing no significant differences between patients with and without bacteriuria before treatment.

### Findings in context of existing evidence

Suprapontine or spinal lesions affect the storage phase and result in reduced bladder capacity and NDO, expressed as involuntary contractions of the detrusor^1^,^12^. These patients often complain about urinary urgency, frequency and incontinence and may suffer from renal failure in the long-term if not appropriately treated^1^,^12^.

As to date, efficacy and safety of intradetrusor onabotulinumtoxinA injections has been reported in >50 studies including some high-level evidence studies^7^,^8^,^21^,^22^,^23^,^24^,^25^. This minimally invasive intervention has become a well-established second-line treatment for patients with refractory NDO to be considered before more invasive therapies such as bladder augmentation, urinary diversion or sacral anterior root stimulation with dorsal rhizotomy^1^,^12^. After regulatory approval of intradetrusor onabotulinumtoxinA injections by the US Food and Drug Administration and the European Union in August 2011 based on the results of two phase 3 studies in patients with multiple sclerosis and SCI suffering from NDO incontinence^7^,^8^, this treatment is expected to increase worldwide, especially as efficacy of repeated injections seems given^20^,^26^,^27^. Regarding antibiotic prophylaxis during this kind of treatment in patients with NDO, evidence-based specific recommendations do not exist at present time. Nevertheless, it is widely used by physicians in daily clinical practice. According to EAU Guidelines on Urological Infections^11^, peri-interventional antibiotic prophylaxis is not generally recommended during cystoscopy and fulguration of small bladder tumours, but during TUR-P and procedures breaching of the bladder mucosa and therefore considered to be clean-contaminated^28^,^29^, always taking additionally into account specific risk factors such as indwelling catheters and bacterial burden^11^. Otherwise neuro-urological patients presenting with asymptomatic bacteriuria should not be treated antimicrobially^12^ in order to avoid significantly more resistant bacterial strains without improvement of outcome. In most previously published studies evaluating safety and efficacy of intradetrusor onabotulinumtoxinA injections in patients suffering from NDO, information about the use of antibiotic prophylaxis is lacking. Schurch *et al*. demonstrated significant improvement of clinical and urodynamic parameters after intradetrusor onabotulinumtoxinA (200 or 300 units) injections compared to placebo in patients with NDO caused by multiple sclerosis or SCI. Injections were performed under antibiotic prophylaxis, administered for an appropriate period of time - but further details were not given^30^. Regarding safety, one of the main adverse events reported in literature is the development of UTI. However, the variability between the studies is wide, after both, treatment and placebo application. Cruz *et al*.^7^ reported a prevalence of UTI in about 22% in the placebo group, 28% in the group treated with 200 units and 38% in the group treated with 300 units onabotulinumtoxinA, respectively. Herschorn *et al*.^21^ showed a UTI rate of 55% in the patients receiving placebo and 57% in patients treated with 200 units onabotulinumtoxinA. In the study by Ginsberg *et al*.^16^, UTI occurred in 34% after placebo and in approximately 50% after treatment. Schurch *et al*.^30^ reported a frequency of UTI of about 14% (placebo group) and 32% (200 units onabotulinumtoxinA group), performing injections under administration of prophylactic antibiotics whereas kind of medication used and duration of applications were not described. However, it should be mentioned that a differentiation between asymptomatic bacteriuria and UTI (or often wrongly defined as asymptomatic and symptomatic UTI) was not made in any of these studies. Mouttalib *et al*.^31^ argued in favour of an antibiotic prophylaxis in patients with refractory NDO undergoing intradetrusor onabotulinumtoxinA injections, since they found a UTI incidence rate of about 7% during the first week after treatment. In contrast, Game *et al*.^32^ described a significant reduction of UTI episodes after injections of 300 units onabotulinumtoxinA into the detrusor in 30 patients suffering from NDO emptying the bladder by clean intermittent self-catheterisation, probably as a result of improved bladder function due to appropriate NDO treatment, especially considering that a direct antimicrobial effect of onabotulinumtoxinA has not yet been shown^33^. In our present study not using antibiotic prophylaxis, UTI occurred in 7% (5/73) of patients with a sterile urine culture and in 5% (9/200) who presented with asymptomatic bacteriuria before intradetrusor onabotulinumtoxinA injections. Therefore, asymptomatic bacteriuria might not have a negative impact on safety of intradetrusor onabotulinumtoxinA injections. In addition, asymptomatic bacteriuria seems not to impair efficacy of this treatment, as we were able to demonstrate improvement in clinical and all urodynamic parameters.

### Implication for practice

Patients with NDO require an extensive and specific workup before embarking on an individualized therapy taking into account the patients’ medical and physical condition as well as their expectations^1^. Based on the data of the present study, antibiotic prophylaxis in patients with asymptomatic bacteriuria undergoing intradetrusor onabotulinumtoxinA injections for refractory NDO seems not to be needed and should be critically scrutinized in order to avoid the risk of selecting antimicrobial resistance. While awaiting the results of well-designed risk-stratification studies, we do not recommend the general use of antibiotic prophylaxis for intradetrusor onabotulinumtoxinA injections but it might be considered in selected cases, for instance in immune-compromized patients.

### Implication for research

Given the heterogeneous nature and management of NDO, it would be of great interest to know which subgroup of patients is at highest risk to develop UTIs after onabotulinumtoxinA treatment and therefore will benefit from an antibiotic prophylaxis. Thus, prospective large-scale multicentre studies are highly warranted to investigate this still unanswered question and to further improve the management of patients with NDO.

### Study limitations

Although we evaluated a well-defined patient population with NDO, there are limitations that should be addressed. Most of our patients suffered from SCI, i.e. patients with other neurological disorders were under-represented. Our unit is part of a highly specialized university SCI centre so that a negative selection bias, i.e. inclusion of more severe cases, cannot to be completely ruled out. In addition, our study was not randomized, i.e. we did not compare the outcome of patients with versus without antibiotic prophylaxis. Nevertheless, the present study was prospective and representative of daily clinical practice.

## Conclusions

Asymptomatic bacteriuria in patients undergoing intradetrusor onabotulinumtoxinA injections for NDO did not affect safety and efficacy outcomes. Thus, antibiotic prophylaxis seems not to be justified and needs to be critically reconsidered, especially taking into account the alarming antibiotic resistance worldwide.

## Additional Information

**How to cite this article**: Leitner, L. *et al*. Antibiotic prophylaxis may not be necessary in patients with asymptomatic bacteriuria undergoing intradetrusor onabotulinumtoxinA injections for neurogenic detrusor overactivity. *Sci. Rep.* **6**, 33197; doi: 10.1038/srep33197 (2016).

## Figures and Tables

**Table 1:**
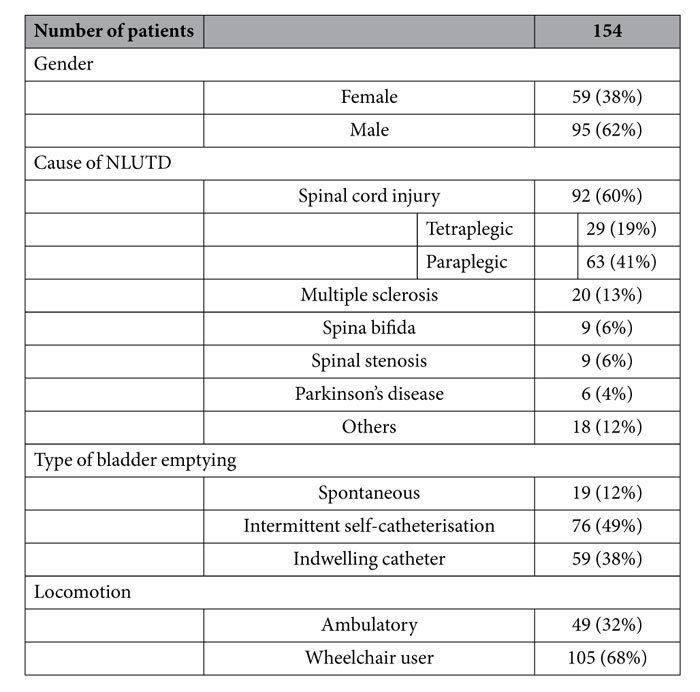
Patients’ characteristics.

**Table 2:** Treatment-related adverse events (per treatment not per patient).

	**Asymptomatic bacteriuria (n = 200)**	**No bacteriuria (n = 73)**
No adverse event	188	68
Gross haematuria	1	0
Urinary tract infection	9	5
Bladder pain	1	0
Autonomic dysreflexia	1	0

**Table 3:** Clinical and urodynamic data.

**Success**	**Pre treatment**	**Pre treatment**	**p**	**Post treatment**	**Post treatment**	**p**
	**Asymptomatic bacteriuria (n = 138)**	**No bacteriuria (n = 54)**		**Asymptomatic bacteriuria (n = 138)**	**No bacteriuria (n = 54)**	
Urinary frequency/24 h	7 ± 4	7 ± 3	0.84	5 ± 1	5 ± 2	0.22
Incontinence episodes/24 h	2 (0–3)	2 (0–3)	0.81	0 (0–0)	0 (0–0)	0.99
MCC [mL]	385 (250–555)	410 (260–595)	0.67	505 (360–715)	535 (510–685)	0.92
Compliance [mL/cmH_2_O]	45 (25–76)	50 (29–74)	0.58	55 (35–110)	71 (39–107)	0.35
pdet_max_ storage [cmH_2_O]	35 (22–54)	40 (20–48)	0.64	19 (11–33)	15 (8–25)	0.05
First DO [mL]	210 (110–325)	230 (120–350)	0.28	325 (190–445)	350 (225–490)	0.28
DO	(138/138)	(54/54)	0.99	(98/138)	(32/54)	0.68
**Failure**	**Asymptomatic bacteriuria (n = 62)**	**No Bacteriuria (n = 19)**		**Asymptomatic bacteriuria (n = 62)**	**No Bacteriuria (n = 19)**	
Urinary frequency/24 h	7 ± 3	7 ± 3	0.57	6 ± 2	6 ± 2	0.67
Incontinence episodes/24 h	1 (1–3)	2 (1–4)	0.27	1 (0–1)	1 (0–2)	0.3
MCC [mL]	330 (195–515)	410 (225–530)	0.24	340 (90–525)	345 (200–450)	0.96
Compliance [mL/cmH_2_O]	32 (19–73)	40 (27–100)	0.15	33 (20–60)	49 (32–100)	0.06
pdet_max_ storage [cmH_2_O]	34 (23–65)	22 (14–48)	0.06	38 (18–58)	34 (21–52)	0.86
First DO [mL]	140 (90–310)	205 (140–370)	0.15	140 (85–270)	190 (105–300)	0.39
DO	(62/62)	(19/19)	0.99	(57/62)	(18/19)	0.12

MCC = maximum cystometric capacity; pdet_max_ storage = maximum detrusor pressure during storage phase; DO = detrusor overactivity; treatment = intradetrusor onabotulinumtoxinA injections.
